# Muscle interconnections in the anterior and posterior arm compartment: a cadaveric case series with possible clinical implications

**DOI:** 10.1007/s00276-023-03209-5

**Published:** 2023-07-19

**Authors:** Konstantinos Natsis, George Tsakotos, George Triantafyllou, Łukasz Olewnik, Nicol Zielinska, Christos Koutserimpas, Trifon Totlis, Maria Piagkou

**Affiliations:** 1 Department of Anatomy and Surgical Anatomy, School of Medicine, Aristotle University of Thessaloniki, Thessaloniki, Greece; 2 Department of Anatomy, School of Medicine, Faculty of Health Sciences, National and Kapodistrian University of Athens, 75 Mikras Asias Str., Goudi, 11527 Athens, Greece; 3 Department of Anatomical Dissection and Donation, Chair of Anatomy and Histology, Medical University of Lodz, Lodz, Poland; 4 Department of Orthopaedics and Traumatology, “251” Hellenic Air Force General Hospital of Athens, Athens, Greece

**Keywords:** Variation, Accessory muscle, Arm, Compression, Brachial artery, Median nerve, Tunnel

## Abstract

**Purpose:**

The report describes four cases of accessory bundles (ABs) or fibers connecting the muscles of the anterior with the posterior arm compartment. The ABs morphology (pure muscular or musculofascial or musculoaponeurotic) is described emphasizing their attachment points, characterized as muscles’ interconnections.

**Materials and methods:**

Four formalin-embalmed donated male cadavers were dissected.

**Results:**

The muscles’ interconnections were unilaterally identified. In the first case, the two ABs originated from the coracobrachialis muscle (CB), received fibers from the biceps brachii (BB), and were inserted into the triceps brachii (TB) medial head. The ABs created an arch over the brachial vessels and the median nerve (MN). In the second case, an accessory musculoaponeurotic structure was identified between CB and TB medial head and extended over the brachial vessels. In the third case, the myofascial ABs between the BB short head and the upper arm fascia, coursed anterior to the MN, the brachial artery, and the ulnar nerve, with direction to the TB medial head. In the fourth case, the three muscular ABs originating from the CB superficial and deep heads, in common with the BB short head, joined the upper arm fascia and the TB medial head and possibly entrapped the musculocutaneous nerve, the MN, and the brachial artery.

**Conclusion:**

ABs or musculoaponeurotic extensions may predispose to complications due to their potential compression on nerves and vessels. Clinicians should consider the possible existence of such bridging variants between muscles, in the differential diagnosis of a patient presenting with ischemia, edema, or MN palsy symptoms.

## Introduction

The anterior muscle compartment of the arm comprises the biceps brachii (BB), the coracobrachialis (CB), and the brachialis (B) muscle. In this compartment, the median nerve (MN), the ulnar nerve (UN), and the brachial vessels (BVs) are typically covered by the muscles. Occasionally, accessory or hypertrophic muscles or accessory heads or accessory bundles (ABs) (purely muscular or myofascial or myo-aponeurotic nature) may pass over the MN, and/or UN and/or the brachial artery (BA) and compress or entrap them [7, 13]. A lot of descriptions have been published about the accessory heads of the anterior muscle compartment of the arm [2, 11, 13], but a limited number of studies [12, 19, 22] are referred to the muscles’ fusion or joining in the anterior and posterior arm compartment with emphasis on the triceps brachii muscle (TB). The formation of muscular or musculoaponeurotic tunnel formations over the arm’s neurovascular elements is sporadic, impeding the determination of a systematic classification pattern and clinical impact.

The present cadaveric series emphasizes the existence of ABs (of muscular or musculofascial or musculoaponeurotic nature) connecting the muscles of the anterior arm with the posterior arm compartment (the so-called muscles’ interconnections). The possible clinical implication is further highlighted.

## Case series

### Methodology

The formalin-embalmed donated male cadavers (aged between 67 and 80 years) were dissected for educational purposes to the Anatomy Department of the Medical School of the National and Kapodistrian University of Athens, to the Anatomy and Surgical Anatomy Department of the Medical School of the Aristotle University of Thessaloniki and to the Department of Anatomical Dissection and Donation of the Medical University of Lodz. The upper limbs’ dissection was performed according to Romanes [17]. Skin, subcutaneous fat, and superficial fascia of the upper limb were dissected, and all muscles of the anterior and posterior arm compartments were exposed from their proximal to the distal attachment. The muscles were carefully examined for a typical or variant attachment, morphology, and innervation. Upper limbs were free of any physical deformity or trauma. The body donation took part through the Body Donation Program after a signed informed consent.

### Cases description

**Fig. 1:**
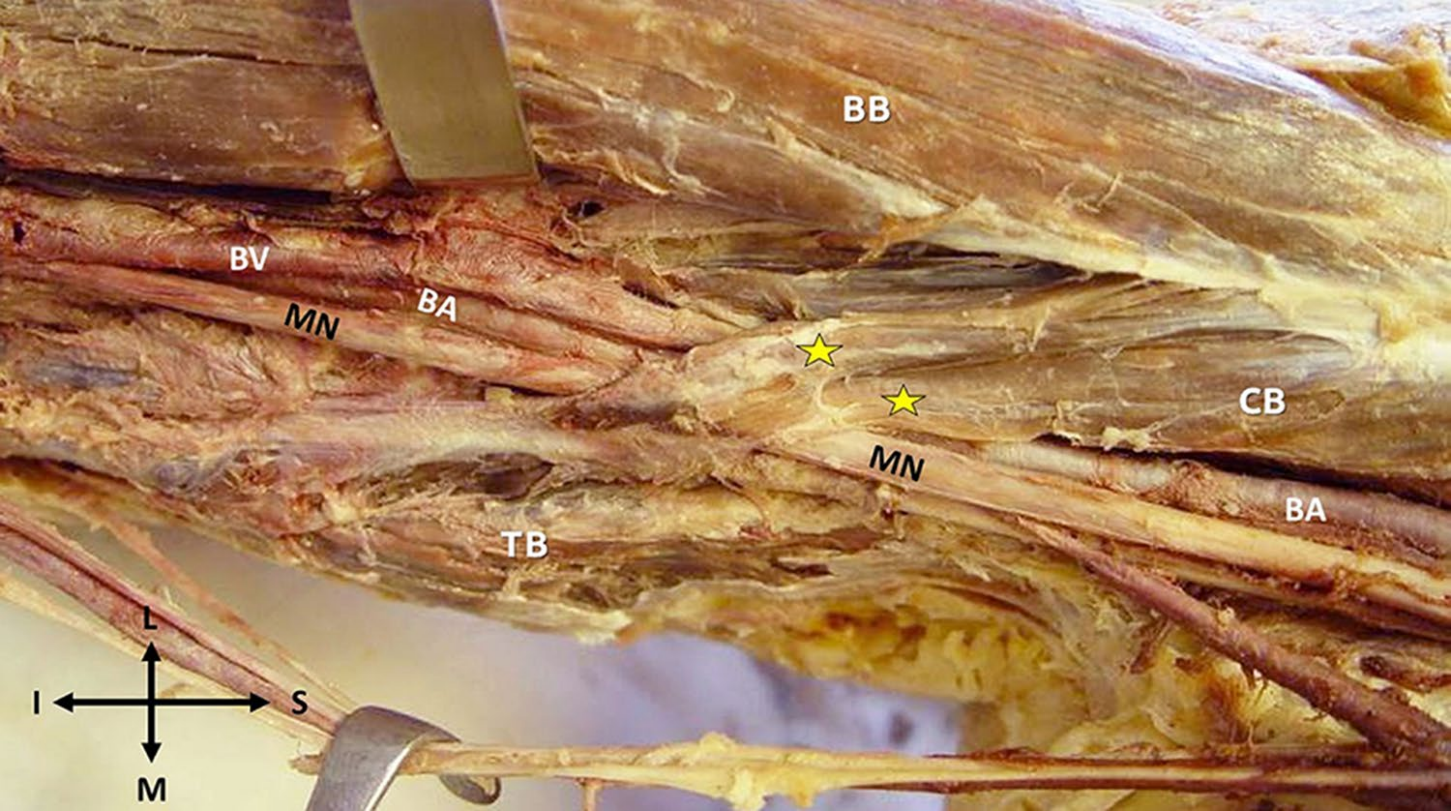
The muscular arch (two yellow asterisks) formed by the fusion of two accessory bundles originating from: 1. The coracobrachialis muscle (CB) and 2. The biceps brachii (BB) short head and joining the medial head of the triceps brachii (TB). The accessory bundles location over the median nerve (MN) and the brachial artery—BA and the brachial vein—BV. The orientation (I—inferior, S—superior, L—lateral, and M—medial) (colour figure online)

Four cases of muscles’ interconnections are summarized in Table 1. **Case 1**: In the right arm of a 67-year-old male cadaver, two muscular ABs were identified. Both bundles originated from the CB, received fibers from the BB, and were inserted into the TB medial head (Fig. 1). The ABs followed an oblique course heading medially and distally and arrayed as an arch over the BVs and the MN. The ABs were innervated by the musculocutaneous nerve (MCN) and supplied by the BA branches.

**Table 1:** Cases’ description by side and points of origin and insertion, as well as the possible occluded structures by the presence of the accessory bundles (ABs)

Cases side R, L	Age (years) Gender	Origin	Insertion	Structures that occluded by ABs
1R	67, M	CB, and fibers from BB	TB medial head	Brachial vessels and MN
2R	72, M	CB	TB medial head	Brachial vessels and MN
3R	80, M	BB short head	Upper arm fascia	BA, MN, and UN
4L	74, M	CB, in common with BB short head	Upper arm fascia and TB medial head	BA, MN, and MCN

*L* left, *R* right, *CB* coracobrachialis muscle, *BB* biceps brachii muscle, *TB* triceps brachii muscle, *MCN* musculocutaneous nerve, *MN* median nerve, *UN* ulnar nerve, *M* males, *ABs* accessory bundles, *BA* brachial artery

**Fig. 2:**
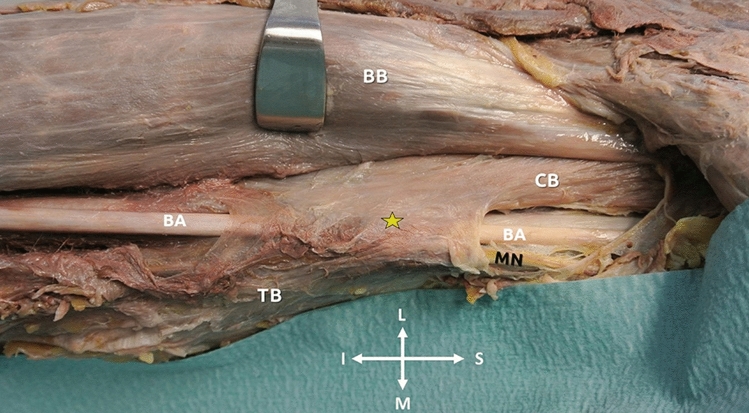
The musculoaponeurotic cover (yellow asterisk) formed by coracobrachialis (CB) and the medial head of the triceps brachii (TB) passing over the brachial artery (BA) and brachial vein and the median nerve (MN). The unyielding borders of the structure resembling to the brachial tunnel as described by Cruveilhier. Orientation (I—inferior, S- superior, L—lateral, and M—medial), BB—biceps brachii muscle (colour figure online)

**Case 2:** In the right upper third of a 72-year-old male cadaver, an accessory musculoaponeurotic tunnel was identified between CB and the TB medial head. The tunnel extended over the brachial vein, and the BA, and had maximum dimensions of 5.2 × 2.7 cm and unyielding borders (Fig. 2).

**Fig. 3:**
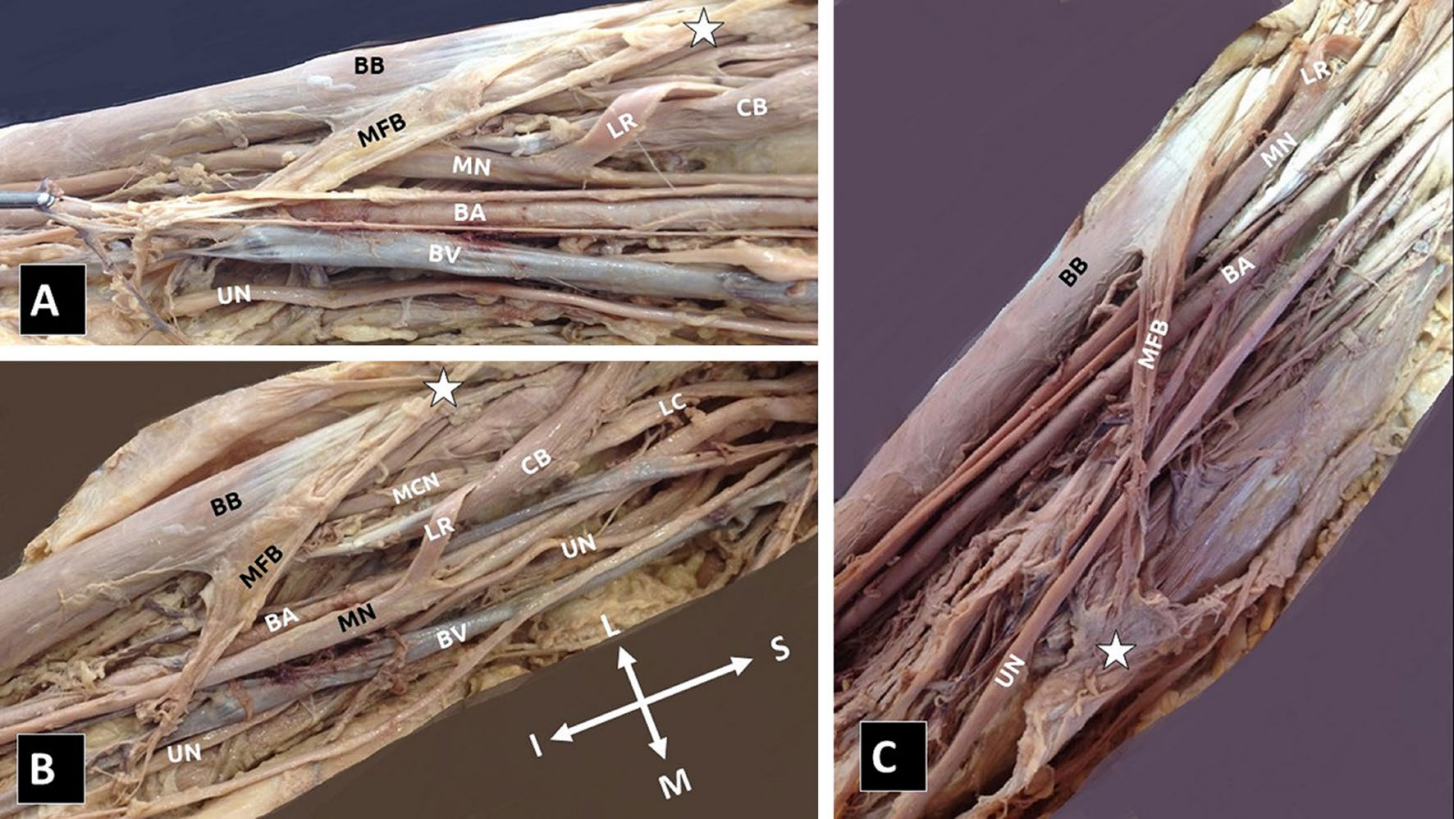
**A**, **B**, **C** The myofascial bundle (MFB) formed by the biceps brachii (BB) short tendon and the upper arm fascia (**A**, **B**, **C**, white asterisks) joining the medial head of the triceps brachii muscle, over the median nerve (MN), brachial artery (BA), and ulnar nerve (UN). **A**, **B** Coracobrachialis muscle (CB) atypical penetration by the musculocutaneous nerve (MCN) and the lateral root (LR) of the MN, BV—brachial vein, LC—lateral cord. The orientation (I—inferior, S—superior, L—lateral, and M—medial) (colour figure online)

**Case 3:** In the right arm of an 80-year-old male cadaver, myofascial ABs were identified between the BB short head and the upper arm fascia. The bundles coursed anteriorly to the MN, the BA, and the UN, with direction to the TB medial head, level of the lower third of the humerus (Fig. 3).

**Fig. 4:**
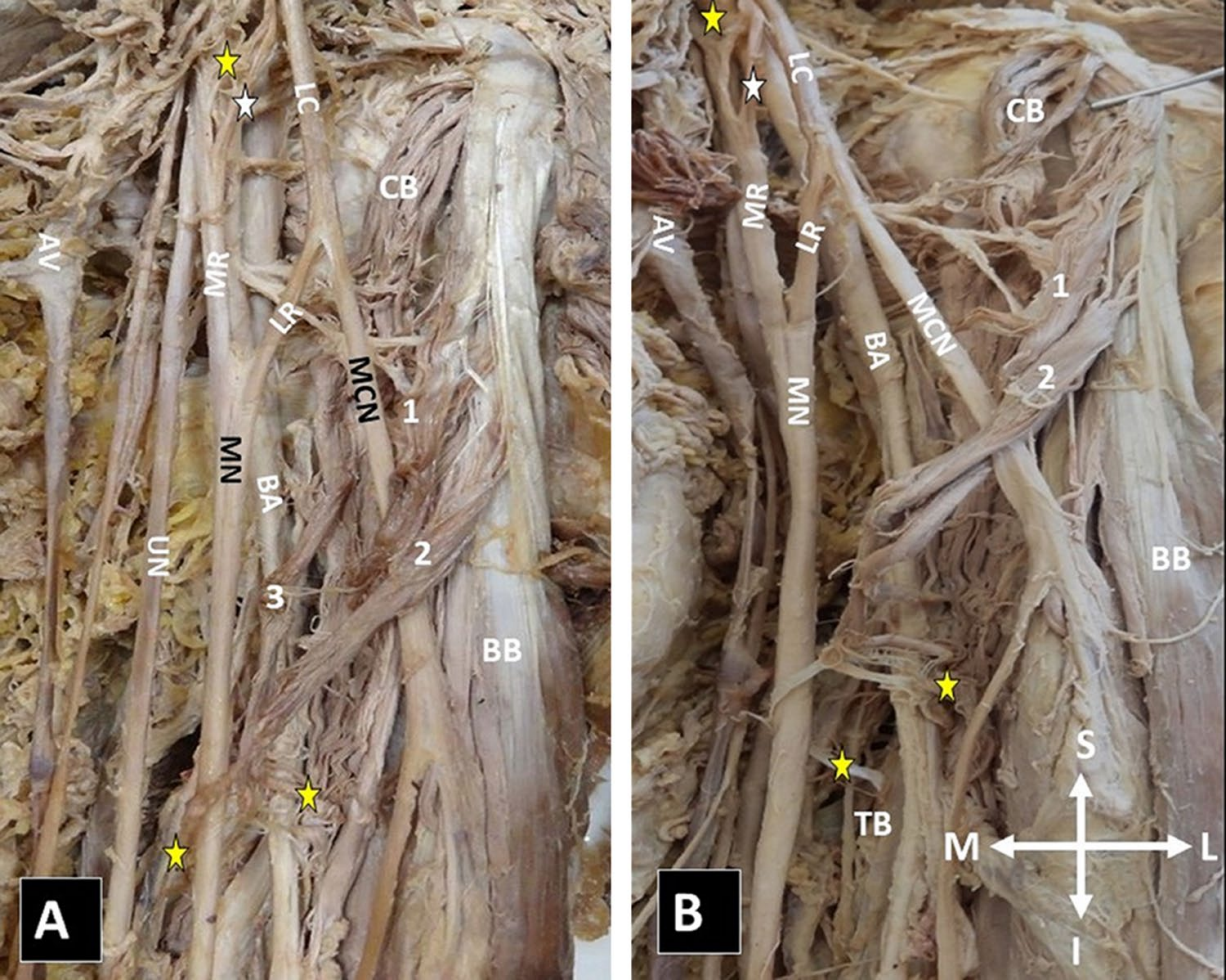
A left-sided three accessory musculofascial bundles (two superficial and a deep) originating from the coracobrachialis (CB) superficial (1, 2) and deep (3) heads, in common origin with the biceps brachii (BB) short head (1, 2) and joining the upper arm fascia, and the medial head of the triceps brachii muscle—TB (**A**, **B** lower yellow asterisks, lateral and medial legs). A possible site of entrapment of the musculocutaneous nerve (MCN, 1, 2), the median nerve (MN, 1, 2), and the brachial artery (BA, 2, 3). **A**, **B** upper yellow and white asterisks—the interconnection of the lateral cord (LC) with the medial cord (upper yellow asterisk) and the medial root (MR) of the MN (white asterisk), AV—axillary vein. The orientation (I—inferior, S—superior, L—lateral, and M—medial) (colour figure online)

**Case 4:** In the left arm of a 74-year-old male cadaver, three muscular ABs (two superficial and a deep) were identified, arising from the CB superficial and deep heads, in common with the BB short head. The ABs joined the upper arm fascia and the TB medial head and possibly entrapped the MCN, the MN, and the BA (Fig. 4). The ABs innervation was provided by the MCN branches, and the arterial supply by the BA branches. The ABs coexisted with an interconnection of the lateral cord of the brachial plexus with the medial cord and the medial root of the MN.

## Discussion

Although a plethora of muscle variants of the anterior arm compartment have been recorded, a limited number of published studies summarized the muscular attachments of the anterior arm to the posterior arm compartment (with a focus on the TB, alternatively characterized as TB interconnections). Thus, no safe conclusions about the prevalence of these muscles’ fusion and possible clinical impact can be conducted.

In the current case series, all identified ABs were not under the form of an additional slip of origin, nor inserted into the ending aponeurotic part of the muscles, so they could not be characterized as accessory heads [5]. The ABs may originate from the B or the BB, (the so-called brachiofascialis muscle), and similar to the third described case of the current series may cross over the MN and/or the BA in the distal arm, leading to MN compression, flattening, and partial perineural thickening [12, 19, 22]. Due to the BA proximity with the MN, the neural entrapment usually accompanies the concomitant BA compression, causing upper limb hypoperfusion [22]. The current second and fourth cases are referred to as a musculoaponeurotic tunnel formation between CB and TB medial head. Similar musculofascial tunnels are described between B and medial intermuscular septum in other studies [12, 15, 22], creating a muscular bridge over MN and BA, with the potential to compress them. The difference between the current cases and other studies is the altered location of neurovascular compression more proximal in the arm, in the current series. In the current first case, the two muscular ABs or slips identified originated from CB and were inserted into TB, during muscles’ contraction could potentially compress on the MN and BA, as they coursed anteriorly to these neurovascular elements according to Nakatani et al. [13].

Although in the current case series, only ABs were identified, it is important to point out other variants that may lead to neurovascular compression, as ABs can cause. The BB, the most superficial muscle of the anterior arm, may have accessory heads that may compress on the MCN [1, 21]. The most frequent variant is the existence of one supernumerary head, the so-called three-headed BB, while more heads may rarely exist [16]. The most common accessory head (the inferomedial head) may originate from the humerus (level of CB insertion), or the B intermuscular septum, and course deep or lateral to the BA and MN, although cases anterior to them have been reported [16]. As regards the B, the commonest variant is the muscle’s division into two or more parts [11]. Insertions of these divisions are highly irregular and versatile and may occur on various parts of the proximal end of the ulna and radius or antebrachial fascia [2]. The B subdivision variant may better explain the tunnels’ formation, through which neurovascular structures may pass and possibly be entrapped. A possible fusion within these parts (layers) may create muscular intra-connections. Considering the embryologic pattern, the aberrant muscular or musculoaponeurotic formations may be remnants of muscle primordia that failed to disappear or fuse to form a unique structure [3, 15]. The B subdivision into two or more parts may result after somatic alterations during embryogenesis or modifications in cell adhesion molecules on the precursor cells [11].

### Clinical impact

Muscle anomalies are difficult to differentiate from soft tissue tumors [7], particularly if the abnormality appears unilaterally, causing asymmetry [9]. The existence of accessory muscles may confuse surgeons intraoperatively and cause neurovascular compression during active movement. The course and direction of the ABs may occasionally create tunnel formations, in which the MN and the BA may be entrapped [12, 15]. The MN during its course, in the upper limb, may pass through several musculoaponeurotic structures, that are constituted by relatively thick layers of connective tissue and have been reported as the main potential sites of neurovascular entrapment, thus explaining the pathophysiology of neuropathy [22]. Mehta et al. [12] described an intramuscular tunnel created by the B, that entrapped brachial vessels and the MN, in the distal arm third. The topography of the above-referred neurovascular structures within the B would possibly render them extremely vulnerable to trauma in case of a fracture of the distal humeral shaft. Since B is a key flexor of the arm, its contraction could compress these neurovascular structures, and its prolonged contraction could lead to paresthesia and numbness. The probability of MN compression proximal to its entrance into the forearm should be taken into consideration. Moreover, remnants of CB variants, such as the CB longus (its accessory tendon), may cross anterior to the MN and BA [6], thus causing neurovascular compression. Gessini et al. [7] reported that the hypertrophic CB longus may be an etiological factor for potential entrapment of the MN, and/or vascular disturbances, due to BA compression.

In the present series, three different muscle variants of the arm are described. Two muscular ABs originating from the CB and BB short head and joining the TB medial head (first case), musculofascial ABs between BB short head and the upper arm fascia (third case), and two musculofascial tunnels (second and fourth case), arising from the CB superficial and deep heads, and joining the upper arm fascia, and the TB medial head. Musculofascial tunnel formations may entrap the deeply situated nerves (MCN and MN), as well as the BA. The muscle or musculofascial tunnels’ variant could lead to diagnostic and therapeutic challenges regarding high MN neuropathy [5]. The meticulous clinical examination may exclude possible areas of entrapment, while morphological details of the nerves supplying the arm muscles are greatly facilitated by magnetic resonance imaging (MRI), which may be utilized in challenging cases [10, 18]. MN entrapment and irritation may lead to high neuropathy [5] including loss of muscle strength, involving all muscles innervated by the nerve, including elbow flexion and forearm supination, as well as sensitivity disruptions, mainly at the forearm’s radial side. Vascular symptoms may also present with homeostasis disturbances of the hand [7], while brachial vessels’ compression may lead to claudication and in more severe cases to edema and/or ischemia. Entrapment of the MN at the anterior arm compartment would lead to motor, as well as sensory deficits. Particularly, it would affect the strength of the superficial volar forearm muscles, including pronator teres, flexor carpi radialis, palmaris longus, flexor digitorum superficialis, and the deep flexors, including flexor digitorum profundus, flexor pollicis longus, and pronator quadratus. Furthermore, the hand muscles, such as the first and second lumbricals, the opponens pollicis, the abductor pollicis brevis, and the flexor pollicis brevis may also be affected. Sensory changes could include the palmar cutaneous branch, as well as the radial 3–1/2 digits. These symptoms could differentiate MN entrapment, at the anterior arm compartment, from carpal tunnel syndrome, anterior interosseous nerve compressive neuropathy, and/or pronator syndrome. In carpal tunnel syndrome, the flexor forearm muscles would show no impairment, at anterior interosseous nerve neuropathy, there would be no sensory deficits, while in pronator syndrome, there would be pain at the proximal volar forearm, sensory changes over the palmar cutaneous branch, and positive Tinel's over the proximal volar forearm. Furthermore, the knowledge of abnormal CB insertion is significant because it can be used as a transitional flap to replace soft tissue deficits in infraclavicular and axillary areas during post-mastectomy reconstruction [8]. The CB flap’s high feasibility may also be exploited for facial reanimation [20], or the own muscle may serve as a guide for anaesthesiologists to locate the MCN [14]. Additionally, keeping in mind that the BB accessory tendon is crucial while performing tendon reconstruction and repair and treating a fracture followed by unusual displacement of the bone fragment [4]. In general, the possibility of a variant should be kept in mind by radiologists when assessing MRI scans, as these features can be misinterpreted [21] and by clinicians when diagnosing or treating patients with weakness or pain in the anterior arm compartment.

## Conclusion

Muscular ABs or slips or the muscles’ aponeurotic extensions may alter the arm’s typical anatomy and predispose to complications, in cases in which their variable arrangement is adequate for a potential compressive action on the nerves crossing the arm and on the superficially located BVs. The passage of neurovascular structures through an actual tunnel further supports the clinical significance of the current cadaveric reports. Clinicians should consider the possibility of such variants, in the differential diagnosis of patients presenting with ischemia, edema, or MN palsy symptoms.

## Data Availability

Data and material related to the report will be available with the corresponding author for further reference.

## References

[1] Aggarwal A, Kaur H, Sahni D, Aggarwal A (2009). Four-headed biceps brachii muscle with variant course of musculocutaneous nerve: anatomical and clinical insight. Int J Anat Var.

[2] Bergman RA, Thompson SA, Afifi AK, Saadeh FA (1988) Muscles. In: Compendium of human anatomical variation Text, Atlas, and world literature. Baltimore, Urban & Schwarzenberg. p 10–11 and 139–142

[3] Cihák R (1972). Ontogenesis of the skeleton and intrinsic muscles of the human hand and foot. Ergeb Anat Entwicklungsgesch.

[4] Daimi SR, Siddiqui AU, Wabale RN, Gandhi KR (2010). Additional tendinous insertion of biceps brachii: a case report. Pravara Med Rev.

[5] Dharap AS (1994). An anomalous muscle in the distal half of the arm. Surg Radiol Anat.

[6] El-Naggar MM, Zahir FI (2001). Two bellies of the coracobrachialis muscle associated with a third head of the biceps brachii muscle. Clin Anat.

[7] Gessini L, Jandolo B, Pietrangeli A (1983). Entrapment neuropathies of the median nerve at and above the elbow. Surg Neurol.

[8] Hobar PC, Rohrich RJ, Mickel TJ (1990). The coracobrachialis muscle flap for coverage of exposed axillary vessels: a salvage procedure. Plast Reconstr Surg.

[9] Kervancioglu P, Orhan M (2011). An anatomical study on the threeheaded biceps brachii in human foetuses, and clinical relevance. Folia Morphol.

[10] Kim YS, Yeh LR, Trudell D, Resnick D (1998). MR imaging of the major nerves about the elbow: cadaveric study examining the effect of flexion and extension of the elbow and pronation and supination of the forearm. Skeletal Radiol.

[11] Loukas M, Louis RG, South G, Alsheik E, Christopherson C (2006). A case of an accessory brachialis muscle. Clin Anat.

[12] Mehta V, Suri RK, Arora J, Kumar H, Yadav Y, Rath G (2010). Crucial neurovascular structures entrapped in a brachial intramuscular tunnel. Rom J Morphol Embryol.

[13] Nakatani T, Tanaka S, Mizukami S (1998). Bilateral fourheaded biceps brachii muscles: the median nerve and brachial artery passing through a tunnel formed by a muscle slip from the accessory head. Clin Anat.

[14] Neal JM, Gerancher JC, Hebl JR, Ilfeld BM, McCartney CJL, Franco CD, Hogan QH (2009). Upper extremity regional anesthesia: essentials of our current understanding, 2008. Reg Anesth Pain Med.

[15] Paraskevas G, Natsis K, Ioannidis O, Papaziogas B, Kitsoulis P, Spanidou S (2008). Accessory muscles in the lower part of the anterior compartment of the arm that may entrap neurovascular elements. Clin Anat.

[16] Rodríguez-Niedenführ M, Vázquez T, Choi D, Parkin I, Sañudo JR (2003). Supernumerary humeral heads of the biceps brachii muscle revisited. Clin Anat.

[17] Romanes GJ (1986). Cunningham’s textbook anatomy.

[18] Rosenberg ZS, Beltran J, Cheung YY, Ro SY, Green SM, Lenzo SR (1993). The elbow: MR features of nerve disorders. Radiology.

[19] Stecco A, Macchi V, Stecco C, Porzionato A, Ann Day J, Delmas V, De Caro R (2009). Anatomical study of myofascial continuity in the anterior region of the upper limb. J Bodyw Mov Ther.

[20] Taylor GI, Cichowitz A, Ang SG, Seneviratne S, Ashton H (2003). Comparative anatomical study of gracilis and coracobrachialis muscle implication for facial reanimation. Plast Reconstr Surg.

[21] Vollala VR, Nagabhooshana S, Bhat SM, Potu BK, Rodrigues V, Pamidi N (2009). Multiple arterial, neural and muscular variations in upper limb of a single cadaver. Rom J Morphol Embryol.

[22] Wadhwa S, Mehra S, Khan RQ, Kapur V (2004). Abnormal musculoaponeurotic tunnel in the arm: possible entrapment of the median nerve and brachial artery with high origin of nerve to pronator teres within tunnel. Clin Anat.

